# Stability and Reproducibility of Radiomic Features Based on Various Segmentation Techniques on Cervical Cancer DWI-MRI

**DOI:** 10.3390/diagnostics12123125

**Published:** 2022-12-12

**Authors:** Zarina Ramli, Muhammad Khalis Abdul Karim, Nuraidayani Effendy, Mohd Amiruddin Abd Rahman, Mohd Mustafa Awang Kechik, Mohamad Johari Ibahim, Nurin Syazwina Mohd Haniff

**Affiliations:** 1 Department of Physics, Faculty of Science, Universiti Putra Malaysia, Serdang 43400, Selangor, Malaysia; 2 Department of Radiology, National Cancer Institute, Putrajaya 65000, Wilayah Persekutuan, Malaysia; 3 Faculty of Medicine, Universiti Teknologi MARA, Sungai Buloh 47200, Selangor, Malaysia

**Keywords:** DWI-MRI, MRI, radiomics, cervical cancer, manual segmentation, semi-automatic segmentation

## Abstract

Cervical cancer is the most common cancer and ranked as 4th in morbidity and mortality among Malaysian women. Currently, Magnetic Resonance Imaging (MRI) is considered as the gold standard imaging modality for tumours with a stage higher than IB2, due to its superiority in diagnostic assessment of tumour infiltration with excellent soft-tissue contrast. In this research, the robustness of semi-automatic segmentation has been evaluated using a flood-fill algorithm for quantitative feature extraction, using 30 diffusion weighted MRI images (DWI-MRI) of cervical cancer patients. The relevant features were extracted from DWI-MRI segmented images of cervical cancer. First order statistics, shape features, and textural features were extracted and analysed. The intra-class relation coefficient (ICC) was used to compare 662 radiomic features extracted from manual and semi-automatic segmentations. Notably, the features extracted from the semi-automatic segmentation and flood filling algorithm (average ICC = 0.952 0.009, *p* > 0.05) were significantly higher than the manual extracted features (average ICC = 0.897 0.011, *p* > 0.05). Henceforth, we demonstrate that the semi-automatic segmentation is slightly expanded to manual segmentation as it produces more robust and reproducible radiomic features.

## 1. Introduction

Cancer is one of the leading causes of significant morbidity and mortality worldwide. In Malaysia, cancer is ranked 4th in causes of death, which contributes to 39.3% as stated in the *Malaysian Study on Cancer Survival*, and cervical cancer is the third most common female cancer [1]. Although the cervical mortality rate in young women is lowest, the incidence rate of cervical cancer has increased among groups of 30- to 60-year-olds [2]. There is a variety of medical imaging modalities for oncological diagnosis and treatment. One of the most vital is Magnetic resonance imaging (MRI) that is extensively used for staging and follow-up diagnoses, especially before the cancer treatment, including subtype tumors in the pelvis region such as rectal, prostate, and cervical cancer [3]. Optimized MRI techniques allow better analysis of tumor biology, superior soft tissue contrast and the tumor in its microenvironment compared to CT and PET. Radiomics MRI recently gained attention in cervical cancer to evaluate the condition of circumferential resection margin, which is associated to the probability of local recurrence [4].

Imaging biomarkers are crucial tools for cancer diagnosis and classification as well as tracking treatment response [5,6]. In current practice, the robust imaging technique known as diffusion weighted magnetic resonance imaging (DW-MRI) utilizes the microscopic mobility of water Brownian motion that correlates to tumor response and lesion aggressiveness. There is a strong chance for DW-MRI to predict treatment efficacy based on pharmacodynamic indicators of drug development. Due to the lack of understanding of DWI-MRI at microscopic level, and no standard of analysis or measurement method having been established, it is crucial to provide a recommendations standard for developing decision support systems with efficacy of DWI-MRI for tumor assessments [5].

The component of radiomic represents high quantitative image features of tumor phenotypes that characterize the volumes of interest. The feature extraction contains information from input images and represents data in lower dimensional space [7,8,9]. This involves a complex mathematical algorithm which describes phenotypes of tumors that are unrecognized and might not be detectable by human observation. There were 20 features selected based on stability, variance of shape and texture features [10]. Results from the study revealed the value of quantitative analysis involved in evaluating the disease on an individual level and the variation of phenotypic of the disease microenvironment [11].

Although radiological texture analysis is capable of classifying diseases, patient stratification and response prediction, there are several factors that influence feature values and may change the research results [12]. Previously, there were several institutions facing major barrier problems of consistency in image acquisition parameters which affect the reproducibility of textural features consisting of pulse sequence, image reconstruction, field strength and different machine parameters. The influence on the repeatability features is also known to be affected by the large variability of image acquisition and feature extraction parameters [13,14].

One of the most significant challenges for radiomics is the accuracy of the tumor segmentation process. Previous research demonstrates that semi-automatic segmentation techniques are selected because they are superior to manual segmentation [14,15]. Manual segmentation is arduous and time consuming compared to semi-automatic segmentation techniques. Research on various segmentation techniques of hepatocellular carcinoma on MRI images and CT scans proves that semi-automatic produces reliable and reproducible radiomic features [16]. Moreover, manual segmentation can result in variability in the segmentation, which is tedious and exhausting for the radiologist and which calls for swifter and more accurate segmentation techniques. Recently, clinical technique segmentation was performed by both human observation and interpretation. This conventional technique was discovered to be time consuming as well as subject to variations. Therefore, the purpose of this research is to evaluate the stability and reproducibility of various segmentation techniques for cervical cancer DWI-MRI images, based on the characteristics of radiomic features.

## 2. Materials and Methods

The contrasted MRI pelvis of cervical cancer with DWI (axial plane) were retrieved retrospectively from the picture archiving communicating system (PACS) system of the Institut Kanser Negara (IKN), Putrajaya, Malaysia. The secondary data were collected randomly from 1 January 2014 to 31 December 2019. This retrospective clinical study was approved by the Medical Research and ethical committee members of the Ministry of Health in Malaysia on 10 August 2022 (ethics initial approval No. NMRR ID-22-01426-OU4 (IIR). Due to the study’s negligible danger to patients, patient consent was waived. We investigated the robustness of characteristics collected from thirty patients in this retrospective study diagnosed with cervical cancer, based on the rule of thumb in the reliability study [17]. Radiologist reports and patient demographic data were collected using random sampling, which is better at eliminating bias and more random for the optimal data gathering outcomes.

### 2.1. MRI DWI-Weighted Cervical Cancer Images

After the screening process, out of 59 patients, only thirty (30) patients (with mean ± SD of age 53 ± 11.33 y/o and weight range from 57.5 ± 7.98 kg) fall within the inclusion criteria: (a) pathologically confirmed cervical cancer from stages II–IV; (b) evaluated by MRI preoperatively; and (c) have DWI-MRI pre contrast imaging. The stages were categorized into three stages: Stage II, Stage III, and Stage IV. All patients were scanned by using 3 Tesla MRI Magnetom Vario (Siemens, Erlangen, Germany). The acquisition protocol for axial DWI images covers the whole uterus and ovaries with varied angulation according to the pathology. The standardized parameter includes slice thickness of 3.0 mm, Time Repetition (TR) 6300 ms, Time Echo (TE) 69.0 ms, Field of View (FoV) 220 mm, base resolution 140 and phase resolution of 100%. The degree of diffusion, b-values were priorly set at 1:50, 2:400 and 3:900 with diffusion scheme monopolar.

This research adopted a single-blinded design, meaning only the researcher was aware of the patient report. The sample was drawn at random and contained stages II–IV cervical cancer diagnoses. Subsequently, two senior radiologists with experience of more than 10 years in clinical reporting of MRI cervical cancer were blinded since they were unaware that their work was carried out on the same sample of images. In order to lessen bias during the picture segmentation procedure, patient identifications were excluded. To prevent patient identities from being disclosed during the segmentation phase, the MRI pelvic images were renamed into a sequence of numbers. To evaluate the reliability of radiomic features generated from segmentation on MR images, three groups are made up to evaluate the first order statistics, shape, and texture. Data radiomic with semi-automatic segmentation were retrieved from volume of interest specified by two separate observers twice using 3D Slicer software, then four independent observers used it to compare it against manual segmentation. Two sets of two segments each were created from the semi-automatic segmentations.

### 2.2. Semi-Automatic Segmentation

**Figure 1:**
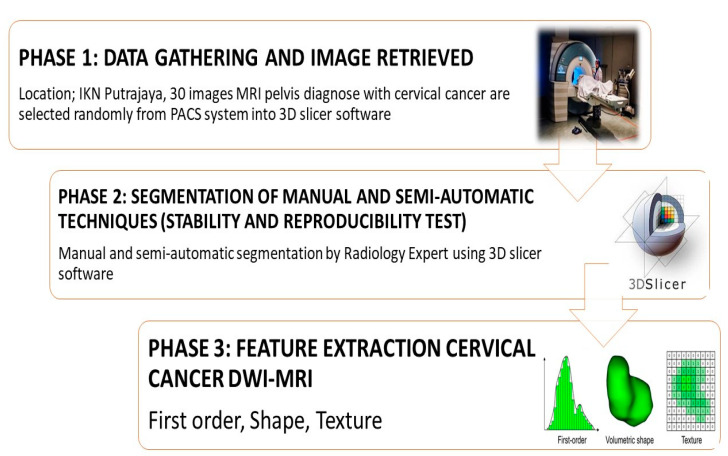
Research framework on stability and reproducibility of cervical cancer DWI-MRI.

For both manual and semi-automatic segmentations we retrieved 662 characteristics using 3D Slicer software (https://slicer.org, accessed on 1 June 2022) (Boston, MA, USA) open-source platform software for 3D medical image analysis used solely for academic research. Moreover, the analysis and comparison of feature extractions for manual and semi-automatic segmentations were evaluated and examined. Despite the short sample size (n = 30), the number of observation sets was investigated to identify a pattern on the retrieved features. In this research a total of 240 segmentations were examined (120 manual segmentations and 120 semi-automatic segmentations). Figure 1 shows the overall research workflow in stability and reproducibility of the cervical cancer DWI-MRI in various segmentation techniques.

**Figure 2:**
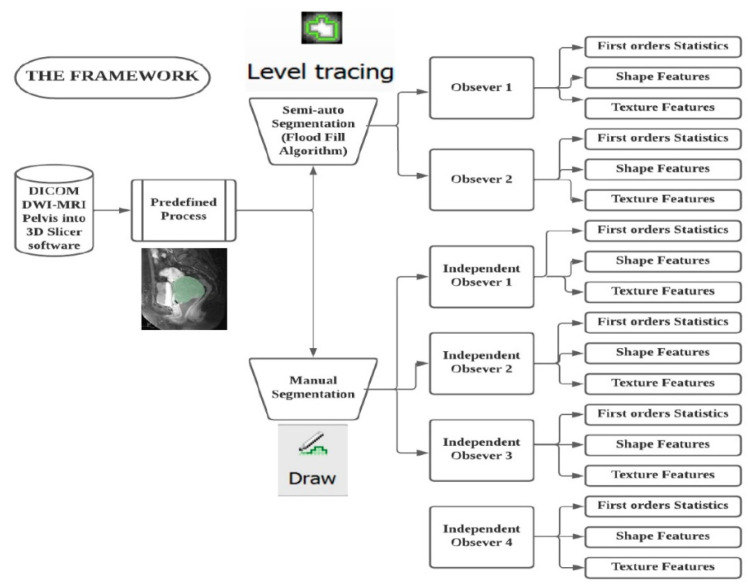
The research process in comparing different segmentation process.

We utilized a flood fill algorithm for semi-automated segmentation. The algorithm was part of an extension that was installed through the extension manager. Two radiologists with experience of more than 10 years were appointed as observers. The observers identified the site of cervical cancer through the DICOM module. Figure 2 illustrates the process of comparing the segmentation technique between manual and semi-auto segmentation. Using a mouse cursor, nodes were appointed around the tumour area. Following that, the flood fill effects were executed, and ROIs were segmented based on the intensity of similar voxels. To identify the connectedness of a region in a multi-dimensional array, the flood fill approach selected intensity voxels that were like the selected node that was specified by users.

**Figure 3:**
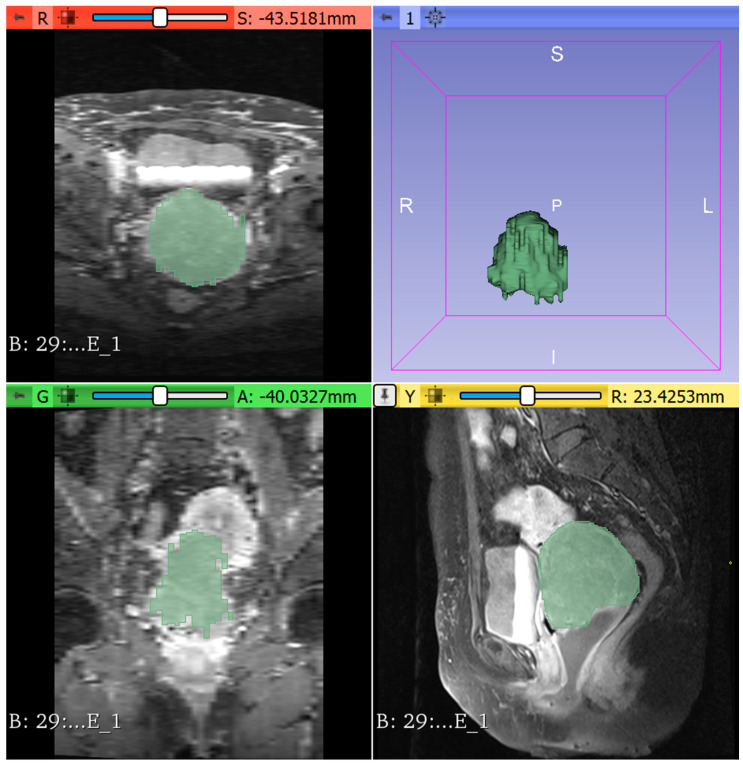
Example of flood-fill algorithm segmentation using 3D slicer software.

Figure 3 shows the effect of flood fill algorithm during the segmentation process. This approach is analogous to the bucket tool in paint applications, which uses different colours to fill related voxels of similar intensity. Choosing the area of interest as the initial point, the algorithm was started. This effect considers pixels linked in four directions to both the first node and the second node. After deciding on the intensity voxels, the computer identified the target node’s route and replaced it with different hues. The neighbourhood size parameter was used to control leakage prevention to other structures under this effect, with voxels of varying intensities and colours. Subsequently, the manual segmentation was placed as the finalisation process to finish the outcome.

### 2.3. Feature Extraction

All segmented image data were analyzed and run through the same application, 3D Slicer under the Radiomics module, to extract imaging characteristics. The mathematical approach based on pixel intensities was used to extract features. We generated 86 radiomic features for MR images from semi-automatic and manual segmentations to aid in measuring tumour characteristics. First order statistics, shape, and texture were the first three groupings of characteristics, and examples of shape features are included in Table 1. Total number of features shape (14), textural characteristics (54) and first order statistics (18) were obtained from the volume of interest.

The first-order statistic which is also known as “tumour intensity” is used to identify the tumour location in an MRI histogram of voxel intensity. The volume properties of the tumour are used to calculate and describe shape features. The py-Radiomics software extracts shape characteristics that are independent of the intensity of the grey levels and can only be computed on the original image. As the wavelet filter is not calculated on derived images it does not apply to form characteristics. Patterns or spatial distributions of voxel intensities were extracted from grey level run-length matrices (GLRLM), grey level co-occurrence matrix (GLCM) and grey level dependence matrix (GLDM). The characteristics were obtained using co-occurrence and run-length matrices by averaging all 13 symmetric directions in three dimensions [18].

### 2.4. Statistical Analysis

Correlations within a class of data are referred to by the intra-class correlation coefficient (ICC). It was estimated to quantify the reproducibility of acquired characteristics. Depending on the experimental circumstance, three distinct types of ICC models can be utilized. Variance estimates were collected for this investigation in order to calculate the ICC for inter-observer segmentations using a two-way mixed effect model of analysis of variance (ANOVA) [6]. The ICC is described by the equation below:

(1) ICC A,1=MSR−MSEMSR+k+1MSg+knMSc−MSE

where *MS_C_* = mean square for columns, *MS_R_* = mean square for rows, *MS_E_* = mean square error, *MS_W_* = mean square for residual sources of variance, *k* and *n* indicate the number of observers and participants occupied.

One-way analysis of variance was used to determine the ICC values for intra-observer segmentation (ANOVA) the following equation defines ICC (C, 1):

(2) $$\mathrm{ICC}\;\left(C,\;1\right)=\frac{MS_{R}-MS_{w}}{MS_{R}+\left(k-1\right)MS_{w}}$$

We assess intra-observer reproducibility by having one observer segment 30 patients in two-month intervals, demonstrating variation in data collected using two different segmentation methods. This also aids in evaluating multiple segmentation algorithms within the same observer initializations. The delineation was completed by several observers using the same methods of segmentation for inter-observer reproducibility, and the degree of agreement between different observers was analyzed. The Wilcoxon rank-sum test with a *p*-value of 0.05 was used to assess the difference in reproducibility for each segmentation. The mean standard deviation was used to express all data. Statistical Package for Social Sciences (SPSS) version 26 was used to conduct overall data analysis.

## 3. Results

**Figure 4:**
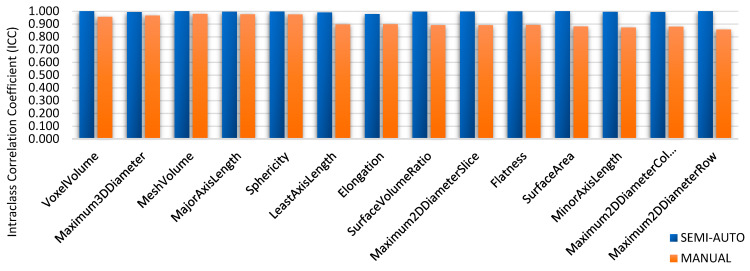
Comparisons of shape-based feature values for the intra-class correlation coefficient (ICC).

**Figure 5:**
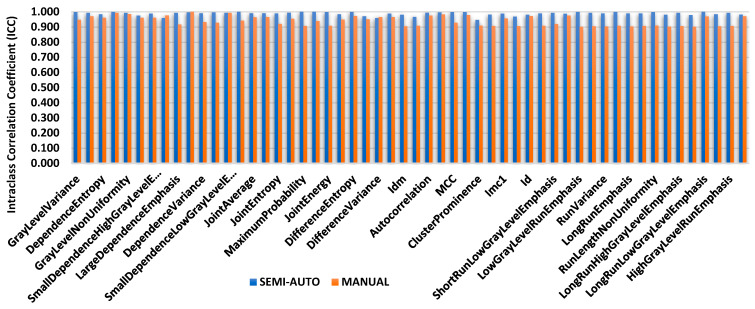
Intra-class correlation coefficient (ICC) comparisons for textural features.

**Figure 6:**
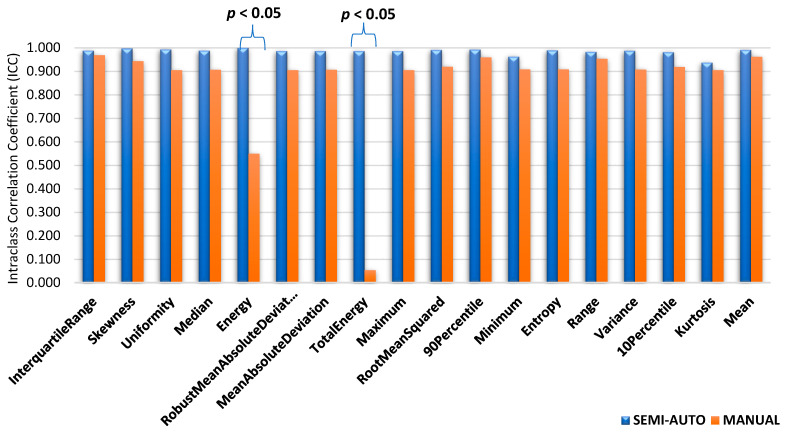
Intra-class correlation coefficient (ICC) value comparisons for first order statistical characteristics for (*p* < 0.05).

**Figure 7:**
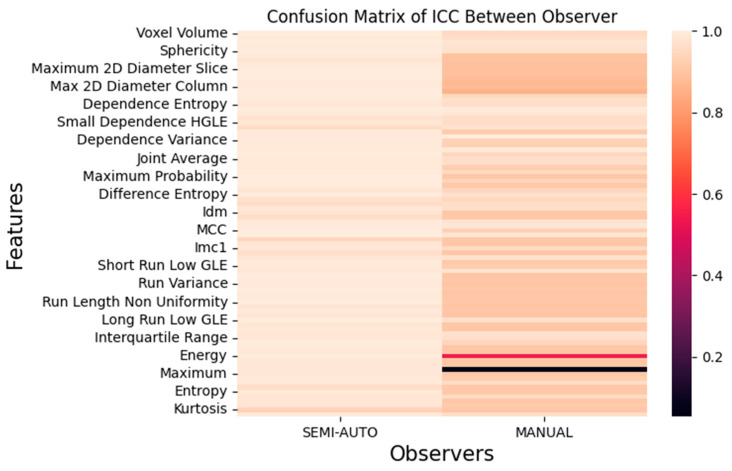
Correlation heatmap between observers.

To evaluate the reliability of radiomic features generated from the segmentation on MR images, three groups made up a total of 86 features. First groups are tumour intensity, shape, and texture. Data radiomic with semi-automatic segmentation were retrieved from the volume of interest specified by two separate observers twice using 3D Slicer software, then four independent observers used it to compare it against manual segmentation. Prior to the methods, the Radiomics Quality Score (RQS) 2.0 obtained were 35.45/36 (98.4%) [19]. The intra-class correlation coefficient (ICC) of shape-based features is shown in Figure 4, Figure 5 and Figure 6; they show the comparisons of ICC in terms of classified characteristics and the first order statistics features between semi-automatic and manual segmentation, respectively. We recognized that semi-automatic segmentation had much higher reproducibility (average ICC = 0.952 ± 0.009, *p* < 0.05) than features derived from manual segmentation (average ICC = 0.897 ± 0.011, *p* > 0.05). Figure 7 presents the correlation heatmap between semi-auto segmentation and manual segmentation. Notably, the semi-auto segmentation shows the extent of the correlation heatmap compared to manual segmentation on both ends of the spectrum in lighter shades.

According to ICC values, extracted features are categorized into three: high reproducibility (ICC ≥ 0.8); medium reproducibility (0.8 ≥ ICC ≥ 0.5); and low reproducibility (ICC < 0.5). Based on 86 features, manual segmentation had 97.67% high, 1.16% medium, and 1.6% low reproducibility. The flood filling effect, which is a result of semi-automatic segmentation, has 100% features in high reproducibility. Table 2 present features derived from reproducibility groups in both segmentations. As a result, Semi-automatic segmentations had better repeatability of the derived characteristics.

**Figure 8:**
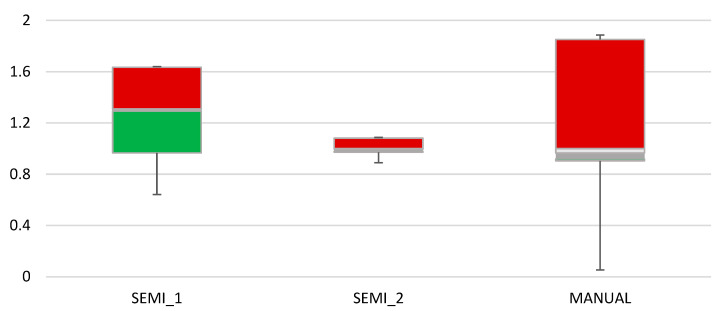
The boxplot of the ICC value reflects the repeatability of radiomic properties across observers.

Each technique’s robustness was assessed by examining the ICC of characteristics collected from inter- and intra-observers. According to Figure 8, inter-observer ICC values from semi-automatic segmentation were found to be high (ICC = 0.976 0.006 and ICC = 0.978, 0.003, respectively). The inter-observer reproducibility is also summarised in Table 3.

## 4. Discussion

Images from DWI-MRI were utilized because they have the possibility of serving as an early surrogate imaging biomarker for the therapy responsiveness indicator [20]. It has been discovered that the quality of input images increases radiomic feature robustness because higher resolution images enhance visual representation for the segmentations [21]. Despite advances in the use of radiomic research, one of the important issues that oncologists face is the robustness and reproducibility of radiomic characteristics retrieved from MR images.

In this research, we utilized 3D-Slicer software to compare the reproducibility and robustness between semi-automatic and manual segmentations, radiomic characteristics of DWI-MRI of cervical cancer images. From the volume of interest, 86 characteristics in total were retrieved and divided into three main groupings (18 of tumour intensity, 14 of shape features and 54 textural features). In accordance with the findings, segmentation that is semi-automatic generates greater ICC values than manual segmentation. The application of a flood filling algorithm in semi-automatic segmentation shows more rhombus and accurate segmentation. According to the uniform color in flood filling algorithm around the region of interest, the tumour lesion was digitized accurately compared to manual segmentation [22]. This algorithm is considered to be the ideal segmentation approach by comparing their accuracy in object selection [23]. Most of the tumour intensity features (first order statistics) result in higher reproducibility compared to manual segmentation (*p* < 0.05). In addition, comparing ICC values in reproducibility between semi-automatic segmentation to conventional segmentation with ICC values, the former had better repeatability with 97% and 78% good in reproducibility. Semi-automatic segmentation results in higher reproducibility compared to manual segmentation, due to less time taken in segmentation and using standard viewing setting for manual segmentation in evaluating intracellular progress of DWI-MRI images. As an outcome, this research suggested to maintain a standard workstation used for image reporting and viewing in clinical settings because it may affect the image interpretation in segmentation of cervical cancer tumors [24]. Additionally, the contouring in clinical target volumes in radiotherapy was heavily dependent on medical imaging, especially in MRI pelvis that provides excellent soft tissue contrast [25,26]. The crucial steps for radiation planning (RT) are the accuracy in delineation of contouring clinical target volume (CTV) from MRI images due to invisibility of microscopic extensions [27]. This problem results in a wide range of contour variability among radiation oncologists depending on their knowledge and experience.

The evaluations of performance between intra- and inter-observer reproducibility were analyses for both segmentations. Figure 4 shows the difference between two observers with clinical experience, especially in the MRI reporting field. Observers’ experience and human interaction may affect the difference in ICC between observers. As a result, features extracted from semi-automatic segmentation proved more reproducible and robust with high ICC values compared to manual, for both intra- and inter-observer segmentation. This indicates that semi-automatic segmentation characteristics tend to be more consistent, reliable, and repeatable. Semi-automatic segmentation based on radiomics features showed a high value in identifying corresponding chemoradiotherapy sensitivity for cervical cancer patients [28]. The contribution from this research may give a higher impact to the patient management in identifying accurate treatment strategies while reducing morbidity and healthcare costs [29].

Furthermore, semi-automatic segmentation of machine-learning algorithms may enhance the predictive performance by measuring the accuracy of image classification in detecting cervical cancer. Compared to the existing methods of manual segmentation, which are time consuming in human resources and monotonous, these result in low and medium variability of interobserver compared to the semi-automatic technique. This research is confirmed by the prior research conducted in the radiation oncologist field on treatment planning of semi-auto segmentation that can significantly reduce their burden and increase inter-observer variability for radiotherapy purposes [30]. Recently, several automated pelvic auto-segmentation procedures have been evaluated, and the advancement of a more effective segmentation method have been proved to apply in the advance phase. The same suggestion was supported by a previous study in rectal cancer; MRI for prostate cancer and other organs have shown that semi-auto segmentation contributes to enhancing medical imaging diagnosis and radiotherapy treatment planning [31].

The limitation of this research is due to insufficient images from other multi center sources for data comparison. DWI-MRI of cervical cancer images were obtained from the center only, and the variability of imaging may affect the radiomic properties. For possible improvements in this research, the next reliability study can take a glance at numerous institutions rather than just single institution.

## 5. Conclusions

This research compared the two approaches’ ability to reproduce radiomic characteristics. Flood fill segmentation, which is semi-automatic, produces more repeatable features with 100% features in high reproducibility, indicating that it could be a preferable alternative to manual segmentation and enhance current diagnostic capability. The overall shape features, texture features and first order statistics for both manual and semi-auto segmentation results had an excellent reproducibility and stability (ICC > 0.9). As a direct consequence, sufficient input from multi center by using this algorithm could be adapted to classification models and prognoses. It should be noted that the study concentrated on the reproducibility and robustness of features generated via semi-automatic and manual segmentation. As an outcome, in process of adapting machine learning, the studies need to be conducted on a larger scale with a larger dataset, so that the application is much more precise and reliable.

## Figures and Tables

**Table 1:** 86 radiomic characteristics were utilising using a 3D-slicer.

Shape Features (n = 14)	GLDM, GLCM & GLRLM Texture Features (n = 14)	First Order Statistics (n = 18)
Voxel_Volume	Gray_Level_Variance	Interquartile_Range
Maximum_3D_Diameter	High_Gray_Level_Emphasis	Skewness
Mesh_Volume	Dependence_Entropy	Uniformity
Major_Axis_Length	Dependence_Non_Uniformity	Median
Sphericity	Gray_Level_Non_Uniformity	Energy
Least_Axis_Length	Small_Dependence_Emphasis	Robust_Mean_Absolute_Deviation
Elongation	Small_Dependence_High_Gray_Level_Emphasis	Mean_Absolute_Deviation
Surface_Volume_Ratio	Dependence_Non_Uniformity_Normalized	Total_Energy
Maximum_2D_Diameter_Slice	Large_Dependence_Emphasis	Maximum
Flatness	Large_Dependence_Low_Gray_Level_Emphasis	Root_Mean_Squared
Surface_Area	Dependence_Variance	90 Percentile
Minor_Axis_Length	Large_Dependence_High_Gray_Level_Emphasis	Minimum
Maximum_2D_Diameter_Column	Small_Dependence_Low_Gray_Level_Emphasis	Entropy
Maximum_2D_Diameter_Row	Low_Gray_Level_Emphasis	Range
	Joint_Average	Variance
	Sum_Average	10 Percentile
	Joint_Entropy	Kurtosis
	Cluster_Shade	Mean
	Maximum_Probability	
	Idmn	
	Joint_Energy	
	Contrast	
	Difference_Entropy	
	Inverse_Variance	
	Difference_Variance	
	Idn	
	Idm	
	Correlation	
	Auto_correlation	
	Sum_Entropy	
	MCC	
	Sum_Squares	
	Cluster_Prominence	
	Imc2	
	Imc1	
	Difference_Average	
	Id	
	Cluster_Tendency	
	Short_Run_Low_Gray_Level_Emphasis	
	Gray_Level_Variance	
	Low_Gray_Level_Run_Emphasis	
	Gray_Level_Non_Uniformity_Normalized	
	Run_Variance	
	Gray_Level_Non_Uniformity	
	Long_Run_Emphasis	
	Short_Run_High_Gray_Level_Emphasis	
	Run_Length_Non_Uniformity	
	Short_Run_Emphasis	
	Long_Run_High_Gray_Level_Emphasis	
	Run_Percentage	
	Long_Run_Low_Gray_Level_Emphasis	
	Run_Entropy	
	High_Gray_Level_Run_Emphasis	
	Run_Length_Non_Uniformity_Normalized	

**Table 2:** The reproducibility groups in features extracted.

Reproducibility Groups	Semi-Automatic	Manual
High (ICC ≥ 0.8)	86 (100%)	84 (97.67%)
Medium (0.8 ≥ ICC ≥ 0.5)	0 (0%)	1 (1.16%)
Low (ICC < 0.5)	0 (0%)	1 (1.16%)

**Table 3:** Radiomic feature ICC of inter-observer analysis.

Features	Original	SEMI_1	SEMI_2	MANUAL
Shape	Voxel_Volume	0.999	0.998	0.955
	Maximum_3D_Diameter	0.986	0.982	0.965
	Mesh_Volume	0.999	0.998	0.979
	Major_Axis_Length	0.998	0.988	0.976
	Sphericity	0.996	0.926	0.974
	Least_Axis_Length *	0.997	0.982	0.896
	Elongation *	0.869	0.937	0.897
	Surface_Volume_Ratio	0.979	0.989	0.89
	Maximum_2D_Diameter_Slice	0.993	0.994	0.89
	Flatness	0.985	0.989	0.891
	Surface_Area *	0.996	0.998	0.88
	Minor_Axis_Length *	0.982	0.988	0.871
	Maximum_2D_Diameter_ Column *	0.983	0.977	0.878
	Maximum_2D_Diameter_Row *	0.996	0.972	0.856
GLDM	Gray_Level_Variance	0.996	0.995	0.907
	High_Gray_Level_Emphasis	0.997	0.983	0.901
	Dependence_Entropy	0.872	0.975	0.901
	Dependence_Non_Uniformity	0.997	0.997	0.903
	Gray_Level_Non_Uniformity	0.998	0.997	0.903
	Small_Dependence_Emphasis	0.936	0.973	0.903
	Small_Dependence_High_ Gray_Level_Emphasis	0.968	0.971	0.903
	Dependence_Non_ Uniformity_Normalized	0.981	0.986	0.903
	Large_Dependence_Emphasis	0.988	0.981	0.903
	Large_Dependence_Low_ Gray_Level_Emphasis	0.989	0.994	0.903
	Dependence_Variance	0.979	0.984	0.903
	Large_Dependence_High_ Gray_Level_Emphasis	0.994	0.989	0.903
	Small_Dependence_Low_ Gray_Level_Emphasis	0.967	0.973	0.903
	Low_Gray_Level_Emphasis	0.998	0.999	0.904
GLCM	Joint_Average	0.981	0.971	0.904
	Sum_Average	0.981	0.971	0.915
	Joint_Entropy	0.973	0.973	0.920
	Cluster_Shade	0.991	0.981	0.954
	Maximum_Probability	1	1	0.907
	Idmn	1	0.997	0.938
	Joint_Energy	0.995	0.997	0.908
	Contrast	0.966	0.976	0.948
	Difference_Entropy	0.988	0.961	0.912
	Inverse_Variance	0.936	0.971	0.951
	Difference_Variance	0.995	0.989	0.905
	Idn	0.683	0.986	0.965
	Idm	0.962	0.972	0.904
	Correlation	0.905	0.972	0.908
	Autocorrelation	0.988	0.989	0.975
	Sum_Entropy	0.945	0.993	0.903
	MCC	0.993	0.995	0.927
	Sum_Squares	0.995	0.993	0.908
	Cluster_Prominence	0.939	0.999	0.909
	Imc2	0.965	0.954	0.907
	Imc1	0.978	0.971	0.906
	Difference_Average	0.991	0.959	0.906
	Id	0.958	0.973	0.902
	Cluster_Tendency	0.974	0.972	0.908
GLRLM	Short_Run_Low_Gray_Level Emphasis	0.983	0.976	0.919
	Gray_Level_Variance	0.972	0.963	0.975
	Low_Gray_Level_ Run_Emphasis	0.998	0.999	0.901
	Gray_Level_Non_Uniformity Normalized	0.998	0.997	0.904
	Run_Variance	0.981	0.963	0.903
	Gray_Level_Non_Uniformity	0.997	0.996	0.908
	Long_Run_Emphasis	0.982	0.966	0.903
	Short_Run_High_Gray_Level Emphasis	0.982	0.966	0.907
	Run_Length_Non_Uniformity	0.994	0.998	0.909
	Short_Run_Emphasis	0.996	0.994	0.902
	Long_Run_High_Gray_Level Emphasis	0.986	0.978	0.905
	Run_Percentage	1	0.969	0.901
	Long_Run_Low_Gray_Level Emphasis	0.999	0.998	0.909
	Run_Entropy	0.997	0.997	0.904
	High_Gray_Level_ Run_Emphasis	0.983	0.976	0.907
	Run_Length_Non_Uniformity Normalized	0.967	0.971	0.901
First Order Statistics	Interquartile_Range	0.979	0.979	0.968
	Skewness	0.974	0.936	0.943
	Uniformity	0.991	0.989	0.904
	Median	0.961	0.978	0.906
	Energy	0.999	0.999	0.549
	Robust_Mean_Absolute Deviation	0.966	0.998	0.904
	Mean_Absolute_Deviation	0.969	0.997	0.906
	Total_Energy *	0.999	0.998	0.053
	Maximum	0.978	0.944	0.904
	Root_Mean_Squared	0.989	0.978	0.919
	90_Percentile	0.999	0.998	0.859
	Minimum	0.994	0.997	0.908
	Entropy	0.998	0.967	0.908
	Range *	0.997	0.991	0.853
	Variance	0.972	0.963	0.907
	10_Percentile	0.947	0.986	0.918
	Kurtosis	0.988	0.948	0.904
	Mean *	0.999	0.982	0.861

* Statistically significant (*p* < 0.05).

## Data Availability

All relevant data are included in the study.
